# Acetylcholinesterase in Biofouling Species: Characterization and Mode of Action of Cyanobacteria-Derived Antifouling Agents

**DOI:** 10.3390/toxins7082739

**Published:** 2015-07-24

**Authors:** Joana R. Almeida, Micaela Freitas, Susana Cruz, Pedro N. Leão, Vitor Vasconcelos, Isabel Cunha

**Affiliations:** 1 CIIMAR/CIMAR—Interdisciplinary Centre of Marine and Environmental Research, University of Porto, Rua dos Bragas 289, Porto P 4050-123, Portugal; E-Mails: up201101518@fc.up.pt (M.F.); sluisa.cruz@gmail.com (S.C.); pleao@ciimar.up.pt (P.N.L.); vmvascon@fc.up.pt (V.V.); isabel.cunha@ciimar.up.pt (I.C.); 2 Department of Biology, Faculty of Sciences, University of Porto, Rua do Campo Alegre, Porto P 4069-007, Portugal

**Keywords:** antifouling, biofouling, cyanobacteria, bioactivity, cholinesterases

## Abstract

Effective and ecofriendly antifouling (AF) compounds have been arising from naturally produced chemicals. The objective of this study is to use cyanobacteria-derived agents to investigate the role of acetylcholinesterase (AChE) activity as an effect and/or mode of action of promising AF compounds, since AChE inhibitors were found to inhibit invertebrate larval settlement*.* To pursue this objective, *in vitro* quantification of AChE activity under the effect of several cyanobacterial strain extracts as potential AF agents was performed along with *in vivo* AF (anti-settlement) screening tests. Pre-characterization of different cholinesterases (ChEs) forms present in selected tissues of important biofouling species was performed to confirm the predominance of AChE, and an *in vitro* AF test using pure AChE activity was developed. Eighteen cyanobacteria strains were tested as source of potential AF and AChE inhibitor agents. Results showed effectiveness in selecting promising eco-friendly AF agents, allowing the understanding of the AF biochemical mode of action induced by different compounds. This study also highlights the potential of cyanobacteria as source of AF agents towards invertebrate macrofouling species.

## 1. Introduction

Biofouling is the process by which a range of micro- and macroorganisms attach to natural and artificial underwater surfaces, constituting a diverse settled community and creating serious problems for the maritime industry worldwide [1]. As virtually all submerged artificial structures (ships, pipelines, fishing devices) are subjected to biofouling, large investments are made worldwide in the removal and also prevention of biofouling species settlement by using antifouling (AF) paints [2,3]. The majority of AF paints currently in use are based on biocidal agents that induce general toxic responses in marine ecosystems. Thus, there is a need to search for alternative non-toxic and environmental friendly AF agents, which might inhibit the settlement of selected biofouling species by non-biocidal mechanisms, acting in more specific signaling targets somehow related with settlement processes [4,5]. On the other hand, searching for settlement-inducing agents for commercially important invertebrate species would also be valuable for aquaculture producing systems purposes.

Compounds that might act as neurotransmission disruptors have been described as both promising AF and fouling-inducing agents to invertebrate species [6]. The settlement signals that invertebrate larvae need to induce metamorphosis initiate a cascade signaling pathway, constituted by a range of neurotransmitters [7]. Particularly, the involvement of the cholinergic system, responsible for modulating motor and sensor functions in synaptic neurons by the maintenance of the neurotransmitter acetylcholine (ACh), has been suggested to play a fundamental role in the permanent attachment of barnacles adhesive larvae [8] and in the induction of settlement in mussels [9,10]. A natural sponge-derived compound (poly-APS) was found to be an effective inhibitor of settlement in the barnacle *Balanus amphitrite*, acting on the cholinergic neurotransmission mechanism [11]. In addition, several neurotransmitters and their derivatives, such as choline, succinylcholine chloride, 3,4-dihydroxyphenylalanine (DOPA) and other catecholamines, have been also reported as inducers of larval attachment and metamorphosis in a range of marine invertebrates [8,12,13], reinforcing that these might be also indicators of fouling-inducing agents. Thus, this leads to consider that AChE activity may be a suitable tool to include in screening approaches for natural AF or fouling-inducing compounds, indicating a potential mode of action for these promising compounds. However, this potential seems to be still underexplored. Thus, the first step to the establishment of a screening method based on cholinesterases (ChEs) activity would be the characterization of the different forms of ChEs (acetylcholinesterase (AChE) and pseudocholinesterases (PChEs)) present in specific tissues of selected biofouling species [14]. ChEs characterization is performed primarily on the basis of substrate specificity and according to their susceptibility to selective inhibitors, as AChE is highly specialised and efficient in degrading the neurotransmitter ACh in the neuromuscular junctions and brain cholinergic synapses [15,16,17]. Concerning the effects, AChE inhibition disrupts nervous system and may cause adverse effects on several physiological functions including respiration, feeding and behaviour [17,18,19]. Invertebrate studies show the presence of multiple genes (polymorphism), four or more in some species, and each one encodes for a different form of ChE [16,20,21]. Given the wide application of ChEs as a biomarker of environmental contamination, its biochemical characterization has been performed in various marine invertebrate species [17,22,23,24,25,26] including in some biofouling constituents as *Mytilus* species [27] and recently in *Pollicipes pollicipes* [28]. *M. galloprovincialis* in particular has been the target of several ChEs characterization studies, usually performed in the gills, the most appropriate organ for biomonitoring studies [24,29,30]. However, the highest AChE specific activity in *Mytilus* sp. was found in the foot [27] which was poorly further investigated. Despite previous results suggest the presence of only one pharmacological form of ChE in *M. galloprovincialis* [29], others show consistency in the presence of three forms of ChE encoded by different genes suggesting a genetic polymorphism [30]. However, the prevalence of AChE activity when compared to PChEs is widely demonstrated in this species [22,24,29,30,31]. Regarding *Pollicipes pollicipes,* ChEs’ characterization in the peduncle was recently performed, showing AChE properties as given by substrate preference and specific inhibitors [28]. Considering that previous characterization studies were conducted based on biomonitoring purposes, there is a need to characterize ChEs forms in specific tissues with a role in organism adhesion, using the same characterization approach in different biofouling species.

Considering this, the aim of this study is to perform the characterization of ChEs in specific tissues of important marine invertebrate species of biofouling communities in the NE Atlantic, mussel *Mytilus galloprovincialis*, goose barnacle *Pollicipes pollicipes* and the acorn barnacle *Perforatus (=Balanus) perforatus* and investigate the role of AChE activity inhibition as an effect and/or mode of action of promising AF compounds*.* To pursue this objective, *in vitro* quantification of AChE activity under the effect of several cyanobacterial strain extracts as potential AF/fouling inducing agents was performed along with *in vivo* AF (anti-settlement) screening tests. Cyanobacteria were selected as source of a wide range of secondary metabolites with recognized bioactivity on distinct biological responses including AF properties [32,33].

## 2. Results and Discussion

### 2.1. Cholinesterase Characterization

#### 2.1.1. *Mytilus galloprovincialis*

ChEs activity was observed in the foot of *M. galloprovincialis* as expected, since a previous study on ChE expression pattern have shown that in *M. edulis*, a congeneric species, the highest ChE activity was found in the foot tissue [27].

**Figure 1:**
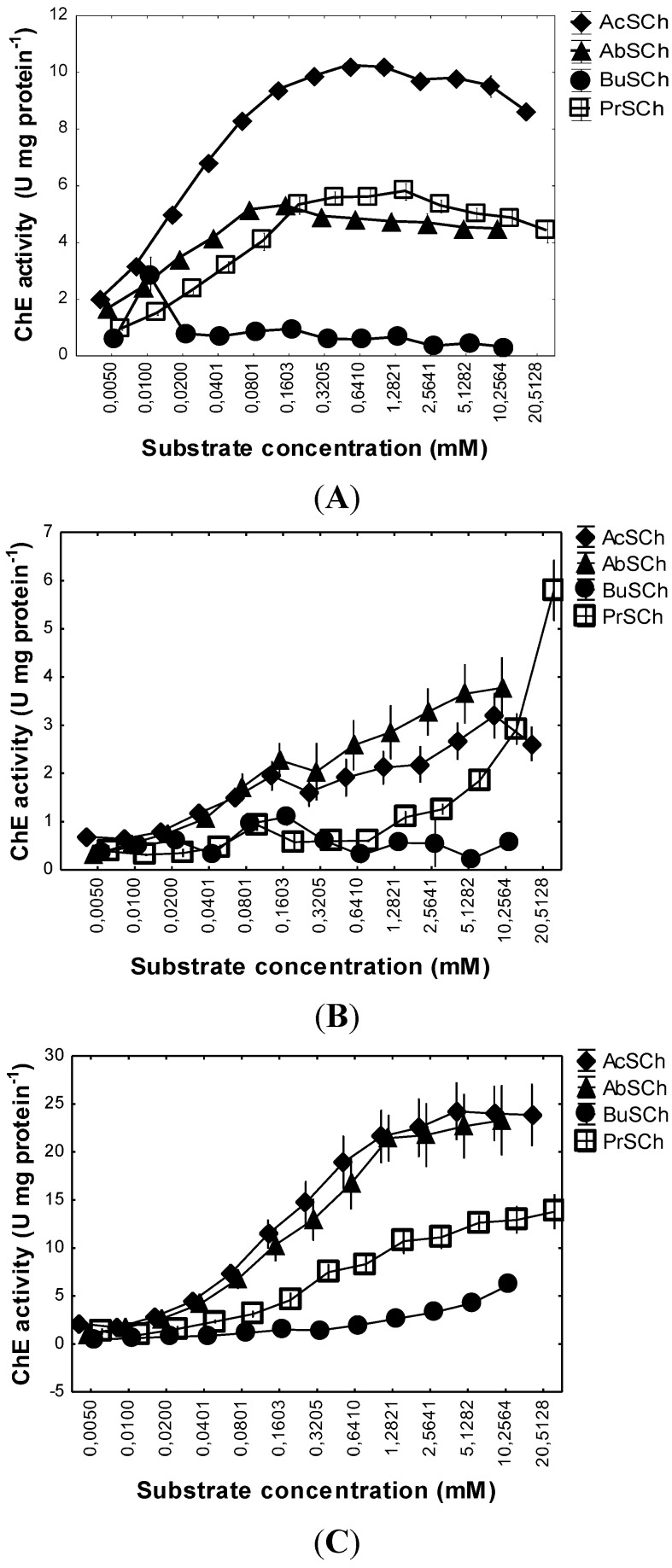
*In vitro* ChE activity on *Mytilus galloprovincialis* foot tissues (**A**), *Pollicipes pollicipes* capitulum soft tissues (**B**) and *Perforatus perforatus* soft whole body (**C**) under a range of concentrations of four different substrates: Acetylthiocholine (AcSCh), Acetyl-β-methylthiocholine (AbSCh), Butyrylthiocholine (BuSCh) and Propionylthiocholine (PrSCh). Values are means with corresponding standard error bars.

Regarding ChEs characterization by substrate preference, *M. galloprovincialis* ChEs degraded preferentially acetylthiocholine (AcSCh), followed by acetyl-β-methylthiocholine (AbSCh) and propionylthiocholine (PrSCh) (Figure 1A), while butyrylthiocholine (BuSCh) was poorly hydrolysed. Inhibition of ChE(s) due to excess of substrate was not found at any of the concentrations tested.

Michaelis constant (*K*_m_) and maximal velocity (*V*_m_) for each substrate were obtained after adjusting the specific activity of each of the four substrates at a range of concentrations, to the best hyperbola through a hyperbolic regression (Table 1; Figure S1), following the Michaelis-Menten kinetics model [34]. *K*_m_ is the substrate concentration needed to reach half of maximum rate (*V*_m_), so the lowest *K*_m_ value indicates that *V*_m_ is reached with less substrate concentration, so having more affinity.

**Table 1:** Kinetic parameters, *K*_m_ (μM) and *V*_m_ (nmol min^−1^ mg protein^−1^), of *Mytilus galloprovincialis*, *Pollicipes pollicipes* and *Perforatus perforatus* ChEs for four reference substrates.

Substrate	*Mytilus Galloprovincialis*		*Pollicipes Pollicipes*		*Perforatus Perforatus*	
	*V*_m_	*K*_m_	*V*_m_	*K*_m_	*V*_m_	*K*_m_
AcSCh	10.51 ± 0.08	22.18 ± 0.75	1.99 ± 0.13	22.97 ± 7.08	27.89 ± 2.03	235.92 ± 44.43
AbSCh	5.81 ± 0.13	14.04 ± 1.12	3.38 ± 0.20	96.81 ± 28.37	23.78 ± 1.94	220.62 ± 51.54
BuSCh	0.85 ± 0.03	2.89 ± 0.88	-	-	6.10 ± 0.46	1418.36 ± 324.90
PrSCh	6.23 ± 0.34	36.07 ± 5.29	3.68 ± 0.53	3716.93 ± 1227.18	13.17 ± 0.46	27912 ± 45.37

The highest *V*_m_ value was obtained for AcSCh (10.51 ± 0.08 nmol min^−1^ mg protein^−1^). AbSCh and PrSCh had a similar *V*_m_ value (5.81 ± 0.13 nmol min^−1^ mg protein^−1^ and 6.23 ± 0.34 nmol min^−1^ mg protein^−1^, respectively) and BuSCh presented the lowest value of *V*_m_ (0.85 ± 0.03 nmol min^−1^ mg protein^−1^). The higher *K*_m_ values were observed with PrSCh (36.07 ± 5.29 μM) followed by AcSCh (22.18 ± 0.75 μM) and then AbSCh (14.04 ± 1.12 μM). The lowest value corresponding to BuSCh (2.89 ± 0.88 μM). This is in accordance with expectancies, since it is a non-specific substrate hydrolysed by all ChEs. The second highest *V*_m_ value occurred for PrSCh and AbSCh, with values close to each other, indicating that the ChE(s) present are able to hydrolyse these two substrates specific of PrSChE and AChE, respectively. For BuSCh, *V*_m_ value was very low, indicating that this substrate is poorly hydrolysed by the ChE(s) present on this species. However, taking into account *K*_m_ values (substrate concentration needed to reach half of maximum rate, *V*_m_), despite BuSCh being poorly hydrolysed, it has the highest affinity for the ChE(s) present. These results suggest that the ChE(s) present on *M. galloprovincialis* foot muscle have predominant hydrolytic characteristics of both AChE and PrChE of vertebrates.

**Figure 2:**
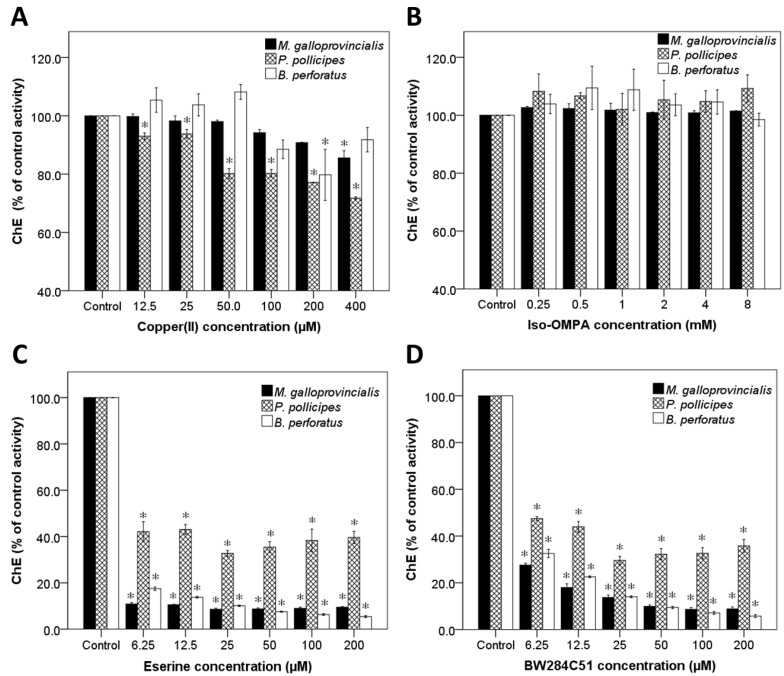
Effects of Cu (II) (**A**), *Iso*-OMPA (**B**), Eserine (**C**) and BW284C51 (**D**) on ChE activity of *Mytilus galloprovincialis* foot, *Pollicipes pollicipes* capitulum soft tissues and *Perforatus perforatus* soft whole body using AcSCh as substrate. Values are the mean of three replicates ± standard error of the mean. * indicates a value significantly different from the control group at *p* < 0.05 (Dunnett test).

Concerning the characterization by ChEs inhibitors, Cu(II), a non-specific inhibitor, only inhibited ChE activity in this species at the higher concentration tested, 400 μM (Figure 2A). Eserine, also a non-specific inhibitor of ChEs, inhibited significantly the ChE activity at all concentrations tested when compared to the control, to values around 10% (Figure 2C). For tetra (monoisopropyl) pyrophosphortetramide (*iso*-OMPA), a specific inhibitor for BuChE, no inhibition of ChE activity was observed at any concentration tested (Figure 2B). Contrarily, for BW284C51, a specific inhibitor of AChE, inhibition of the ChE activity was observed at all concentrations tested (Figure 2D), as evidenced by the lowest observed effect concentration (LOEC) and no observed effect concentration (NOEC) values (Table 2).

Results show that neither Cu(II) nor *iso*-OMPA are capable of inhibiting *M. galloprovincialis* ChE activity, suggesting that these ChE(s) do not have typical characteristics of vertebrates BuChE. These results are in good agreement with the results obtained with the different substrates, as BuSCh was the most poorly hydrolysed substrate. On the other hand, eserine and BW284C51 both inhibit extensively ChEs’ activity on this species. Comparing these two inhibitors, and considering that eserine inhibits all ChEs, and BW284C51 only inhibits AChE, it is evident, at low inhibitor concentrations, that there are other enzymes that hydrolyse AcSCh, most likely other esterases (CoEs). This suggests that about 9% to 10% of the ChE activity observed, with AcSCh as substrate, is probably due to CoEs activity. Conversely, at high concentrations (>25 μM) of eserine and BW284C51, there were no significant differences between ChE activity with those inhibitors, suggesting that ChE(s) present on this species have inhibitory characteristics of vertebrates AChE.

**Table 2:** NOEC and LOEC values for the three species, *Mytilus galloprovincialis*, *Pollicipes pollicipes* and *Perforatus perforatus* with the inhibitors: Cu (II), Eserine and BW284C51.

Inhibitor	*M. Galloprovincialis*		*P. Pollicipes*		*P. Perforatus*	
	NOEC	LOEC	NOEC	LOEC	NOEC	LOEC
Cu (II)	200 μM	400 μM	<12.5 μM	12.5 μM	100 μM	200 μM
Eserine	<6.25 μM	6.25 μM	<6.25 μM	6.25 μM	<6.25 μM	6.25 μM
BW284C51	<6.25 μM	6.25 μM	<6.25 μM	6.25 μM	<6.25 μM	6.25 μM

In *M. edulis* foot, the highest ChEs activity was also observed using AcSCh as substrate, being PrSCh the second most hydrolysed [27]. Moreover, despite that the enzyme activity was inhibited more than 90% by eserine, BW28451 led to inhibition as well, and the activity was not affected by *iso*-OMPA [27], which is in accordance with the results from the present study.

A previous study, using the enzyme phosphatidylinositol-specific phospholipase C (PI-PLC) digestion, followed by electrophoresis, and characterization by inhibition techniques, suggests that *Mytilus* spp. have only one pharmacological form of ChE [29]. Considering this, the ChE present shall be only one, but with enzymatic characteristics of more than one isoenzyme present in vertebrates, according to the present results.

#### 2.1.2. *Pollicipes pollicipes*

*P. pollicipes* ChEs activity was similar for all substrates at low concentrations (<0.04 mM) (Figure 1B). From 0.04 mM, AcSCh and AbSCh show higher activities as compared to BuSCh and PrSCh that maintained lower activities at all concentrations tested (except for PrSCh at 20 mM). Inhibition of ChE(s) due to excess of substrate does not seem to occur at any of the concentrations tested. The hyperbolic regressions of ChE activity on substrate concentration for the four substrates showed that the higher *V*_m_ values were obtained with AbSCh (3.38 ± 0.20 nmol min^−1^ mg protein^−1^) and PrSCh (3.68 ± 0.53 nmol min^−1^ mg protein^−1^), and the lowest value occurred with AcSCh (1.99 ± 0.13 nmol min^−1^ mg protein^−1^). The highest *K*_m_ value was observed with PrSCh (3716.93 ± 1227.18 μM), followed by AbSCh (96.81 ± 28.37 μM) and, the lowest value was observed with AcSCh (22.97 ± 7.08 μM) (Table 1; Figure S2). BuSCh did not adjust to hyperbolic regression and, for that reason it was not possible to obtain the kinetic parameters, *K*_m_ and *V*_m_ for this substrate (Table 1). These results suggest that AcSCh is the preferred substrate, having the lowest *K*_m_ value. That was expected since it is a non-specific substrate hydrolysed by all ChEs families. Despite one of the highest values of *V*_m_ had been obtained for PrSCh, ChE(s) present showed the lowest affinity for this substrate (highest *K*_m_ value), indicating that this substrate is not the preferred one for the ChE(s) present on this species. AcSCh displayed the lowest *V*_m_ although ChE(s) had the highest affinity for it (lowest *K*_m_ value). For AbSCh, it was observed a relatively low *K*_m_ and one of the highest values of *V*_m_. BuSCh did not adjust to hyperbolic regression due to the low activity observed at all concentrations, suggesting that this substrate is poorly hydrolysed by ChE(s). ChE(s) present in this species were able to hydrolyse both AbSCh and PrSCh suggesting that ChE(s) have hydrolytic characteristics similar to both AChE and PrChE from vertebrates.

Regarding ChE inhibitors, Cu(II) (Figure 2A) caused a small inhibition at all concentrations tested (NOEC < 12.5 μM; LOEC = 12.5 μM, Table 2). Eserine, a non-specific inhibitor of ChEs, caused inhibition at all concentrations tested, of about 50% activity as compared to the control (Figure 2B). *Iso*-OMPA, specific for BuChE, caused no inhibition at any concentration tested (Figure 2C). In the case of BW284C51 (Figure 2D), specific for AChE, inhibition was observed at all concentrations tested (NOEC < 6.25 μM; LOEC = 6.25 μM). This shows that *iso*-OMPA does not inhibit ChE activity, at the concentration range tested, and Cu(II) only inhibits it slightly. On the other hand, eserine and BW284C51 both inhibit extensively ChE activity. Comparing these two inhibitors, considering that eserine inhibits all ChEs and BW284C51 only inhibits AChE, and that there were no significant differences between their activities at any inhibitor concentration tested, it is suggested that all ChEs present on this species, if more than one, have inhibition characteristics of vertebrates’ AChE. The remaining activity observed suggests that there are others enzymes that hydrolyse AcSCh, most likely CoEs. This leads us to believe that about half of the activity observed in this species is probably due to CoEs. Similarly to the results from this study, ChE(s) activity in the peduncle of the same species also showed preference for AcSCh and PrSCh as substrate, and poor BuSCh hydrolysis capacity [28]. Regarding inhibitors, it was observed that *iso*-OMPA did not inhibit these ChE(s), contrarily to eserine and BW284C51 that widely inhibit their activity. However, previous results indicate that BW284C51 inhibits these ChEs more effectively than eserine, contrarily to our results, where no significant differences between eserine and BW284C51 inhibition levels were found at all concentrations tested [28]. This suggests that ChEs present in *P. pollicipes* capitulum soft tissues and in peduncle muscle are not exactly the same isoenzymes.

#### 2.1.3. *Perforatus perforatus*

*P. perforatus* ChE activity, using different substrates, showed that the highest activity was observed with AcSCh and AbSCh, followed by PrSCh, and finally BuSCh, which had the lowest activity at all concentrations tested (Figure 1C). Inhibition of ChE(s) due to substrate excess was not observed at any of the concentrations tested. The hyperbolic regressions of ChE activity on substrate concentration, for the four substrates, showed that the highest *V*_m_ was obtained for the substrates AcSCh (27.89 ± 2.03 nmol min^−1^ mg protein^−1^) and AbSCh (23.78 ± 1.94 nmol min^−1^ mg protein^−1^), followed by PrSCh (13.17 ± 0.46 nmol min^−1^ mg protein^−1^), being the lowest value observed for BuSCh (6.10 ± 0.46 nmol min^−1^ mg protein^−1^) (Table 1; Figure S3). The highest *K*_m_ value was found for BuSCh (3716.93 ± 1227.18 μM). Lower values were obtained for AcSCh (235.92 ± 44.43 μM), AbSCh (220.62 ± 51.54 μM) and PrSCh (279.12 ± 45.37 μM). Similarly to what was observed for the other species, results show that ChE(s) in this species have the highest affinity for AcSCh, a non-specific substrate hydrolysed by all ChEs. AbSCh had a *V*_m_ value close to AcSCh and *K*_m_ values were also very close to each other, suggesting that ChE(s) of this species have also a higher preference for AbSCh. PrSCh presented *K*_m_ and *V*_m_ values close to the former two substrates, suggesting that the ChE(s) present show similar affinity for the three substrata. Contrarily, BuSCh is poorly hydrolysed given its low *V*_m_ and high *K*_m_, which indicate low affinity for the enzyme. The results suggest that the ChE(s) of this species have predominant hydrolytic characteristics of both AChE and PrChE from vertebrates, as it was the case of the two former species.

Regarding ChE(s) inhibition, Cu(II) presented low inhibition and only at 200 μM (NOEC = 100 μM) (Figure 2A; Table 2). Eserine inhibited ChE activity at all concentrations tested to values around 10% of the control (Figure 2B). *Iso*-OMPA, the specific inhibitor for BuChE, did not inhibit significantly ChE activity at any concentration tested (Figure 2C). BW284C51 caused ChEs inhibition at all concentration tested, although at low concentrations (6.5–50 mM), the inhibition was significantly lower than observed with eserine. The inhibition results show that neither Cu(II) nor *iso*-OMPA are capable of inhibit the ChEs activity. On the other hand, eserine and BW284C51 both extensively inhibits ChEs activity. Comparing these two inhibitors, and considering that eserine inhibits all ChEs, and BW284C51 only inhibits AChE, at the two higher concentrations (100 and 200 μM) there were no significant differences between the activities, suggesting that all ChEs presents on this species have inhibition characteristics of vertebrates’ AChE. However, the significant differences in the remaining activity observed after using eserine or BW284C51 at lower concentrations suggests that there are others enzymes that hydrolyse AcSCh, most likely CoEs, at a rate about 6%–7% of the total activity observed. Identical results were also observed in this study for *M. galloprovincialis* and *P. pollicipes*. Also, in the grass shrimp, *Palaemonetes pugio*, ChEs present have the properties of vertebrates AChE, hydrolysing both AcSCh and AbSCh; the hydrolysis of BuSCh was minimal; and the ChEs were inhibited by eserine and BW286C51 but not by *iso*-OMPA [26]. Concerning ChEs’ characterization of the amphipod *Echinogammarus meridionalis* and the shrimp *Atyaephyra desmarestii,* similar results were also obtained [35]. This suggests some conservation in the ChE(s) present in these invertebrate species.

### 2.2. Antifouling and AChE Activity Tests

**Figure 3:**
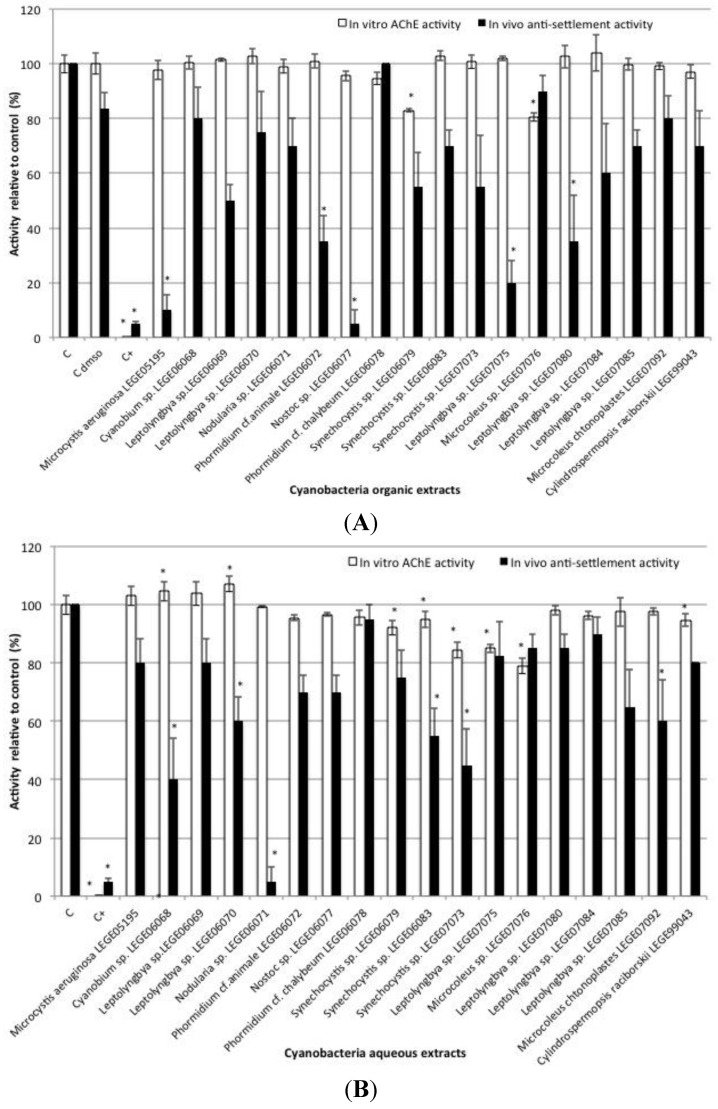
*In vitro* AChE activity and *in vivo* *Mytilus galloprovincialis* plantigrade larvae anti-settlement activity, induced by organic (**A**) and aqueous (**B**) crude extracts of selected strains of cyanobacteria. * indicates a value significantly different from the control group (*C*_dmso_ and *C*, respectively) at *p* < 0.05 (Dunnett test).

Results from the *in vivo* antifouling (anti-settlement) tests using mussel plantigrade larvae exposed to crude extracts of 18 different cyanobacterial strains showed significant bioactivity in some organic and aqueous extracts (Figure 3). Organic extracts from strains *Microcystis aeruginosa* LEGE05195, *Phormidium* cf. *animale* LEGE06072, *Nostoc* sp. LEGE06077, *Leptolyngbya* sp. LEGE07075 and *Leptolyngbya* sp. LEGE07080 significantly inhibited the settlement of plantigrade larvae (*F* = −73.75, *p* < 0.001; *F* = −48.75, *p* = 0.019; *F* = −78.75, *p* < 0.001; *F* = −63.75, *p* = 0.001 and *F* = −48.75, *p* = 0.019, respectively) when compared to DMSO control. No significant differences were found between DMSO control and the negative control with filtered seawater (*F* = 2.0, *p* = 0.05). Regarding aqueous extracts, significant settlement inhibition was observed for *Cyanobium* sp. LEGE06068 (*F* = −60.0, *p* < 0.001), *Leptolyngbya* sp. LEGE06070 (*F* = −40.0, *p* = 0.02), *Nodularia* sp. LEGE06071 (*F* = −95.0, *p* < 0.001), *Synechocystis* sp. LEGE06083 (*F* = −45.0, *p* = 0.006), *Synechocystis* sp. LEGE07073 (*F* = −55.0, *p* < 0.001), *Microcoleus chtonoplastes* LEGE07092 (*F* = −40.0, *p* < 0.001) when compared to filtered seawater control. Positive control (CuSO_4_) was highly responsive to prevent mussel plantigrade larvae settlement (*F* = −83.75 *p* < 0.001). No mortality was observed in any of the conditions tested including the positive control.

Considering the first part of this work demonstrating that the ChEs present on the selected tissues of three important biofouling species used have mainly hydrolytic characteristics of AChE and are inhibited by specific inhibitors of AChE, the *in vitro* screening test was based on pure AChE activity. Inhibition of AChE activity *in vitro* by cyanobacteria organic crude extracts was observed for *Synechocystis* sp. LEGE06079 (*F* = −16.97, *p* < 0.001), and *Microcoleus* sp. LEGE07076 (*F* = −15.56, *p* < 0.001) compared to DMSO control (Figure 3A). Among aqueous crude extracts, *Synechocystis* sp. LEGE06079 (*F* = −7.89, *p* < 0.001), *Synechocystis* sp. LEGE06083 (*F* = −5.39, *p* = 0.007), *Synechocystis* sp. LEGE07073 (*F* = −15.78, *p* < 0.001), *Leptolyngbya* sp. LEGE07075 (*F* = −15.21, *p* < 0.001), *Microcoleus* sp. LEGE07076 (*F* = −21.33, *p* < 0.001) and *Cylindrospermopsis raciborski* LEGE99043 (*F* = −5.61, *p* < 0.001) caused significant inhibition of AChE activity (Figure 3B); and *Cyanobium* sp. LEGE06068 and *Leptolyngbya* sp. LEGE06070 (*F* = 5.21, *p* = 0.01; *F* = 6.76, *p* < 0.001, respectively) caused AChE activity induction, compared to filtered seawater control. Positive control (eserine) was highly responsive regarding AChE activity inhibition (*F* = −100.22, *p* < 0.001).

In this study, *in vivo* anti-settlement responses and *in vitro* potential effect responses based on a neurotransmission-related parameter (AChE activity) involved in the processes of invertebrate settlement [8] were obtained. Both tests were performed in response to aqueous and organic crude extracts of cyanobacteria that are known to produce a wide diversity of secondary metabolites with several bioactive properties [32], and which are good candidates as a source of AF compounds [33]. The results obtained from the *in vivo* anti-settlement tests indicated that selected cyanobacterial strains showed significant bioactivity on both organic and aqueous crude extracts, confirming their great AF potential towards invertebrate settlement. Concerning the *in vitro* AChE activity tested, results were also responsive by discriminating cyanobacteria extracts with AChE inhibition and induction potential.

Considering the combined responses of the two tests, two cyanobacterial aqueous extracts (*Synechocystis* sp. LEGE06083 and *Synechocystis* sp. LEGE07073) showed bioactivity for both anti-settlement and AChE activity inhibition. This suggests that the two activities might be related, and that the AChE activity disruption observed with the crude extracts of these strains might be responsible for the unsuccessful adhesion. Crude extracts of the genus *Synechocystis* were also found to induce acute toxicity to *Artemia salina* nauplii, to affect embryonic development of *Paracentrotus lividus* and to completely inhibit the embryogenesis in *M. galloprovincialis* [36]. Antimicrobial and cytotoxic activities were also found from marine *Synechocystis* sp. extracts by inducing apoptosis in eukaryotic cells and causing inhibition of Gram-positive bacteria [37]. Still, results from the present study indicated potential to inhibit AChE activity of another organic extract of *Synechocystis* sp. LEGE06079, however with no relation with settlement impairment. So, these results seem to indicate the presence of bioactive metabolites on these strains, and probably indicating that different promising compounds with AChE activity inhibition mode of action were acting when different activities were found. As it was expected, not all settlement inhibition was explained by an effect on AChE activity, as modes of action of AF compounds are diverse. Several cyanobacterial extracts analyzed were highly competent in the prevention of settlement with no effect on AChE activity. It is the case of all organic extracts that significantly inhibited settlement, and for instance, the aqueous extract of *Nodularia* sp. LEGE06070 with 95% of settlement inhibition and no effect on AChE activity.

Another interesting finding of this work was an opposite effect on AChE activity (induction rather than inhibition) by aqueous extracts of *Cyanobium* sp. LEGE06068 and *Leptolyngbya* sp. LEGE06070, associated with significant *in vivo* anti-settlement activity. This might indicate that anti-settlement is being induced by a different metabolite, other than the one that is modulating AChE activity, or alternatively by a combination of different metabolites that may be working synergistically.

Some of the cyanobacteria extracts that showed anti-settlement potential in this study were previously found to produce effects on other different endpoints. Namely, organic extracts of *Leptolyngbya* sp. LEGE07075 and *Leptolyngbya* sp. LEGE07080 were able to inhibit the growth of other cyanobacteria (*Synechocystis salina*) [38], and also compromised the normal development of *P. lividus* pluteus larvae at higher extract concentration (1.6 mg mL^−1^) [39]; the organic extract of *Nostoc* sp*.* LEGE06077 affects the growth of other marine cyanobacteria species like *Synechocystis salina*, freshwater species such as *Microcystis aeruginosa* and the microalgae *Nannochloropsis* sp. [38]; aqueous extract of *Leptolyngbya* sp. LEGE06070 was previously found to induce toxic effects on *Artemia salina* [39]; *Cyanobium* strains were found to inhibit microalgae (*Nannochloropsis* sp.) and bacterial (Pseudomonas putida NB3L) growth, and also induced a decrease in *P. lividus* larvae growth [40]. This also demonstrates some potential of the secondary metabolites produced by these cyanobacteria strains to induce metabolic alterations in invertebrate larvae.

Considering this, further studies on the chemical purification and elucidation of the active principles of the selected bioactive strains, with AF properties, should be conducted to possibly obtain new natural effective AF agents. In addition, the combination of the *in vitro* AChE activity with the *in vivo* anti-settlement tests in the screening approach seems to be valuable in the assessment of promising AF compounds; one assessing a selected phenotypic target (settlement), integrating the effect of many possible compounds on various pathways, and the other assessing the effects on a specific mechanism that may be involved in settlement inhibition, the inhibition of AChE.

## 3. Experimental Section

All experiments were conducted in accordance with ethical guidelines of the European Union Council (Directive 2010/63/EU) and the Portuguese Agricultural Ministry (Portaria nr. 1005/92 of 23 October) for the protection of animals used for experimental and other scientific purposes.

### 3.1. Invertebrates Provenience and Transport

This study has focused on three main biofouling invertebrate species of the Portuguese coast, *M. galloprovincialis* (mussel), *P. pollicipes* (goose barnacle) and *P. perforatus* (acorn barnacle). All species are abundant in the intertidal zone, occur all year round and are easy to capture. They have also in common an initial planktonic period in their life cycles, after which settlement and metamorphosis into adult sessile organisms occurs.

Specimens were collected in the intertidal rocky shore, during low tide in Memória beach, Matosinhos, Portugal (41°13′59″ N; 8°43′28″ W), and immediately transported to the laboratory in controlled conditions.

### 3.2. Tissues Preparation and Protein Quantification

Selected tissues from each invertebrate species containing the adhesive organs (mussels) and/or the majority of nervous tissues (barnacles) were isolated in refrigeration. The anterior part of foot muscle, in the case of *M. galloprovincialis*, the whole capitulum for *P. pollicipes*, and the whole soft tissues for *P. perforatus*. *M. galloprovincialis* 2 to 3 feet were pooled per sample. For *P. pollicipes*, the capitula of 2 to 3 animals, and for *P. perforatus* the soft tissues of 5 animals were homogenized on ice in 1 mL of K-phosphate buffer 0.1 M (K_2_HPO_4_ and KH_2_PO_4_ from Sigma-Aldrich, St. Louis, MO, USA) at pH 7.2 using an ultraturrax (Heidolph SilentCrusher M, Schwabach, Germany) and, when necessary, an ultrasonic homogenizer (Bandelin SONOREX RK 100H, Berlin, Germany) was used for short cycles to avoid warming up and degradation of the sample. Homogenates were centrifuged at 6000 rpm for 3 min at 4 °C (VWR MicroStar 17R, Radnor, PA, USA). Supernatants were stored at −80 °C until further analyses.

Protein determination of samples was performed in triplicate using bovine serum albumin (BSA) (Sigma-Aldrich, St. Louis, MO, USA) as standard [41] in a microplate: 0.25 mL of the Bradford reagent (diluted 5 times in ultra-pure water) were added to 0.01 mL of tissue; after an incubation period of 15 min at 25 °C, the absorbance was read at 600 nm in a BioTek Synergy HT microplate reader (BioTek Instruments, Winooski, VT, USA). All samples were then diluted to a concentration of 0.3 mg mL^−1^.

### 3.3. Cholinesterases Characterization According to the Preference for Specific Substrates and Response to Specific Inhibitors

For ChE characterization according to the preference for a specific molecule upon which the enzyme acts (substrate), three specific substrates were used: acetyl-β-methylthiocholine (AbSCh) (SIGMA A3271), specific for AChE; butyrylthiocholine (BuSCh) (SIGMA B3253), specific for butyrylcholinesterases (BuChE); propionylthiocholine (PrSCh) (SIGMA P2880), specific for propionylcholinesterases (PrChE); and a non-specific substrate, acetylthiocholine (AcSCh) (SIGMA A5751). Reaction solutions were prepared for each substrate at 13 concentrations ranging from 6.0 μM to 24.62 mM [17,25] with 5,5′-dithiobis-(2-nitrobenzoic acid) (DTNB) 0.32 mM and sodium bicarbonate 0.57 mM in phosphate buffer 0.1 M at pH 7.2. ChEs activity was determined at 412 nm for 15 min at 25 °C according to the Ellman’s method [42], adapted to microplate using a volume of 0.05 mL of tissue sample (supernatant) and 0.25 of reaction solution. Three independent samples constituted by pooled tissues as previously described were analysed, and activity was measured in quadruplicate of each pooled sample. For each substrate concentration, a blank was prepared containing 0.25 mL of reaction solution and 0.05 mL of phosphate buffer.

Three inhibitors were used for ChE characterization according to the response to selective inhibitors: eserine (SIGMA E8375), a non-specific reversible inhibitor; BW284C51 (SIGMA A9013), an AChE specific reversible inhibitor; *Iso*-OMPA (SIGMA T1505), a BuChE specific inhibitor; and Cupper (II), a non-specific inhibitor. *Iso*-OMPA solutions were prepared in 100% ethanol, while for the other inhibitors ultrapure water was used. Test concentrations of inhibitors ranged from 6.25 to 200 μM of eserine and BW284C51, 0.25 to 8 mM of *iso*-OMPA and 12.5 to 400 µM on assays with copper(II) [17,25,43]. For each inhibitor, six concentrations were tested and, as in the previous experiment, three independent replicates were made, each one in quadruplicate. For this experiment, incubation solutions were prepared with the supernatants of each species and the inhibitor, at the concentration range mentioned before. All incubation solutions receive the inhibitor at the same time (or in the shortest period of time), and were allowed to incubate for 30 min at 25 °C. A control was prepared for each replicate, containing the sample without inhibitor, to determine the normal ChE activity of the sample. This control solution was also submitted to 30 min of incubation. ChEs activity was determined as previously described. The reaction solution contained AcSCh 0.47 mM as substrate. Blanks (phosphate buffer) were prepared for all inhibitor concentrations.

### 3.4. Cyanobacteria Culture and Extracts Production

Eighteen cyanobacteria strains from Portuguese estuaries, intertidal rocky beaches and freshwater systems (LEGE culture collection) were cultured and up-scaled in aerated Z8 medium [44] under laboratory conditions at 25 °C, light/dark cycle of 14/10 h and light intensity of approximately 25 × 10^−6^ E m^−2^ s^−1^. After 60 to 90 days of growth, the cyanobacterial cells were collected and lyophilized. The biomass from each cyanobacterial strain was repeatedly extracted with warm (<40 °C) CH_2_Cl_2_/MeOH (2:1) and the solvents removed *in vacuo* and/or under a N_2_ stream [45].

After the organic extraction, the remaining biomass was subjected to aqueous extraction (ultra-pure water), decanted and centrifuged at 4600 rpm for 15 min. The resulting supernatant was freeze-dried, weighed and stored at −20 °C.

Just before the tests, organic extracts were dissolved (30 mg mL^−1^) in dimethyl-sulfoxide (DMSO) and aqueous extracts in ultra-pure water.

### 3.5. In vivo Antifouling Tests

These tests aim to investigate the ability of organic and aqueous extracts of cyanobacteria strains to inhibit the settlement of *M. galloprovincialis* plantigrade larvae, assessed by the production/non production of mussel adhesive structure (byssus threads).

Test solutions were obtained by dilution of the cyanobacteria extracts stock solutions in filtered seawater to a concentration of 30 µg mL^−1^. *M. galloprovincialis* plantigrade larvae were exposed to test solutions in 24-well microplates for 15 h in the darkness, to maximize byssal threads production. Four well replicates were used per condition with five larvae per well. A negative control with filtered seawater was included in all tests, as well as a positive control with 5 μM CuSO_4_ (a potent AF agent). A second negative control (C DMSO) was used only in the organic extracts assays, including filtered seawater with 0.1% DMSO, to control the effect of the solvent.

At the end of the exposure period, the presence/absence and number of byssal threads produced by each individual was assessed for all the conditions.

### 3.6. In vivo AChE Activity

These *in vitro* screening tests aim to test the hypothesis that some extracts of cyanobacteria strains act as AF agents through ChE inhibition.

AChE activity was determined as previously described [42], using acethylthiocholine as substrate, pure enzyme acetylcholinesterase from *Electrophorus electricus* Type V-S (SIGMA C2888, E.C. 3.1.1.7) and crude extracts of cyanobacteria as inhibition/induction agents. Briefly, 250 μL of the reaction solution containing 30 mL of phosphate buffer, 1000 μL of the reagent dithiobisnitrobenzoate (DTNB) 10 mM (acid dithiobisnitrobenzoate and sodium hydrogen carbonate in phosphate buffer) and 200 μL of acetylcholine iodide 0.075 M was added to 50 μL of pure acetylcholinesterase enzyme (0.25 U/mL) and 3 μL of each cyanobacteria extract (final concentration of 30 μg mL^−1^) in quadruplicate. The optical density was measured at 412 nm in a microplate reader during 5 min at 25 °C. A negative control with ultra-pure water instead of extract was included in all assays as well as a positive control with 20 mM eserine (a potent AChE inhibitor). A second negative control (C DMSO) was used in the organic extracts assays including ultra-pure water with 0.1% DMSO, to account for the possible effect of the solvent. Blank conditions were also included containing 50 μL of phosphate buffer instead of pure AChE.

### 3.7. Data Analysis

All data concerning ChEs activity characterization, *in vivo* anti-settlement tests and *in vitro* AChE activity were analysed using a one-way analysis of variance (ANOVA) followed by a multi-comparisons Dunnett test (*p* < 0.05). When ANOVA presuppositions were not met in the data, a non-parametric test, the Kruskal-Wallis on ranks, followed by the Dunn’s test, were used. ChEs activity kinetics for the different substrates was obtained according to Michaelis-Menten model [34]. A non-linear regression was used to fit data directly to the best hyperbola and the kinetic parameters, *K*_m_ and *V*_m_, for each substrate, were obtained through a hyperbolic regression analysis using the software STATISTICA 12. *K*_m_ (μM) is the concentration of substrate at which half the active sites are filled, providing a measure of the substrate concentration required for significant catalysis to occur. *V*_m_ (nmol min^−1^ mg protein^−1^) represents the number of substrate molecules converted into product by the enzyme at full substrate saturation [34]*.*

In the case of data from ChEs’ characterization using inhibitors, Dunnett’s test was used to compare each treatment with the control and to determine the values of the no observed effect concentration (NOEC) and the lowest observed effect concentration (LOEC) [46]. The software IBM SPSS Statistics 21 was used for statistical analysis.

## 4. Conclusions

This study allowed to establish that ChEs present on *M. galloprovincialis* foot, *P. pollicipes* capitulum soft tissues and *P. perforatus* whole soft tissues have predominantly hydrolytic characteristic of vertebrates AChE and PrChE, and are inhibited by specific inhibitors of vertebrates AChE. This suggests that ChE isoenzymes of these three species have characteristics of both vertebrates AChE and PrChE. Considering this and the role of AChE in larval settlement, *in vitro* AChE activity inhibition was used along with an *in vivo* anti-settlement test in a screening approach for promising AF agents using cyanobacteria strains as a source of bioactive secondary metabolites. Results from this study contribute to a better understanding of the nature and identity of these biofouling invertebrate species ChEs that will be helpful to further studies using ChEs activity on these species. Also, the optimization of the *in vitro* ChEs assays will be helpful for further AF and fouling-inducing screening approaches, and for the understanding of modes of action that are responsible for the AF activity induced by different compounds, including cyanobacteria-derived products. The combination of *in vivo* and *in vitro* responses proved to be a good strategy in the search for eco-friendly AF compounds.

## Supplementary Materials

Supplementary materials can be accessed at: http://www.mdpi.com/2072-6651/7/8/2739/s1.
