# Supplementary material for: Acetylcholinesterase in Biofouling Species: Characterization and Mode of Action of Cyanobacteria-Derived Antifouling Agents
Source: Toxins (Basel). 2015 Jul 24;7(8):2739–56. doi: 10.3390/toxins7082739 (PMC4549721; doi:10.3390/toxins7082739)
Supplement: Supplementary file 1 [file toxins-07-02739-s001.pdf]

Supplementary Materials

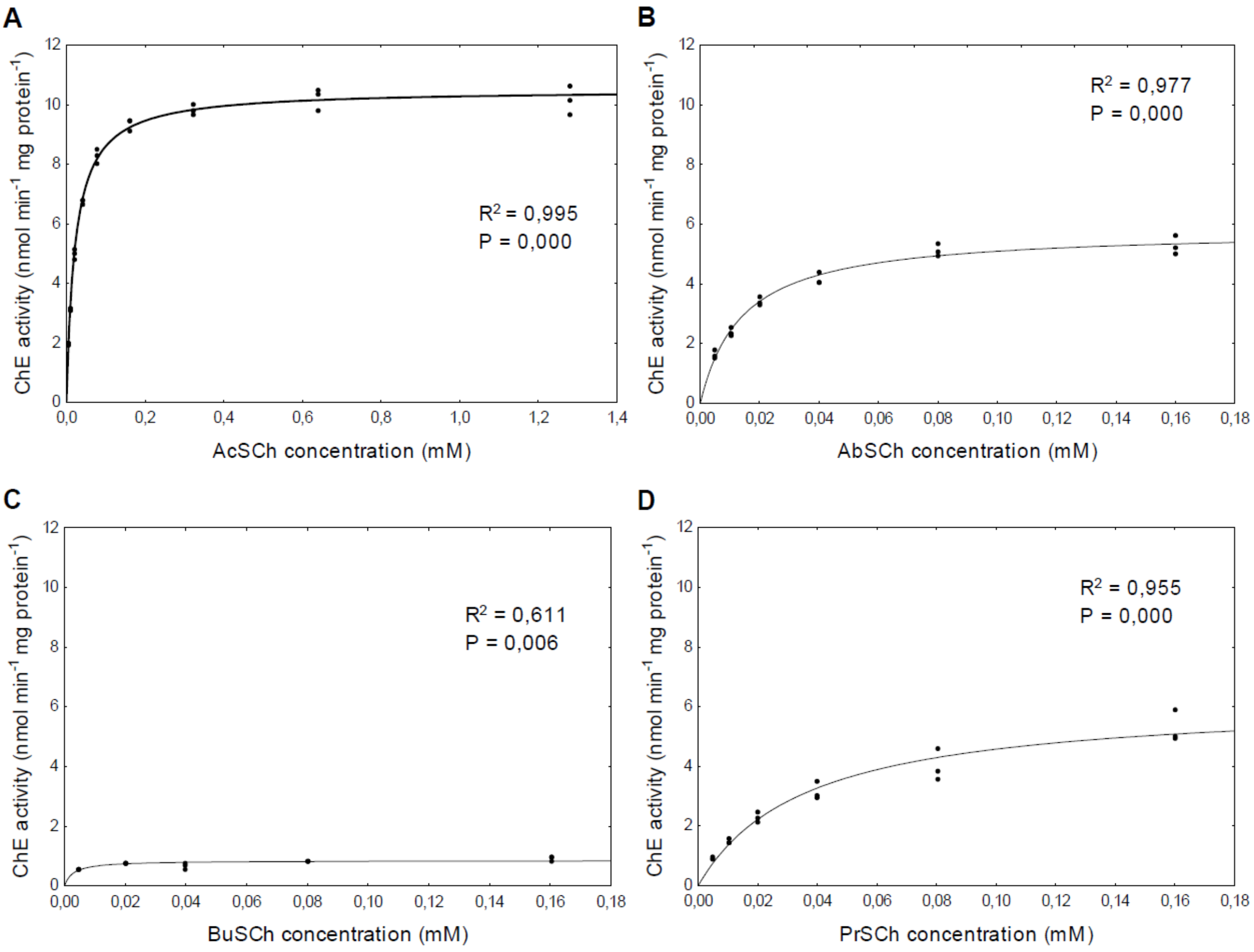

Figure S1. Hyperbolic regression of the specific ChE activity of Mytilus galloprovincialis foot on substrate concentration, for of the four substrates tested. (**A**): AcSCh; (**B**): AbSCh; (**C**): BuSCh; (**D**): PrSCh in foot homogenates.

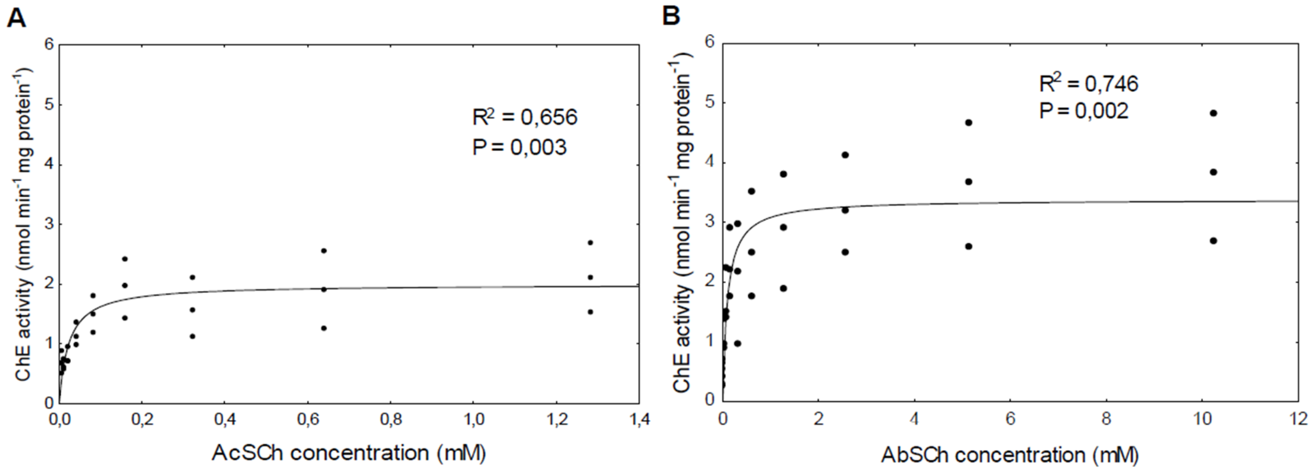

Figure S2. *Cont.*

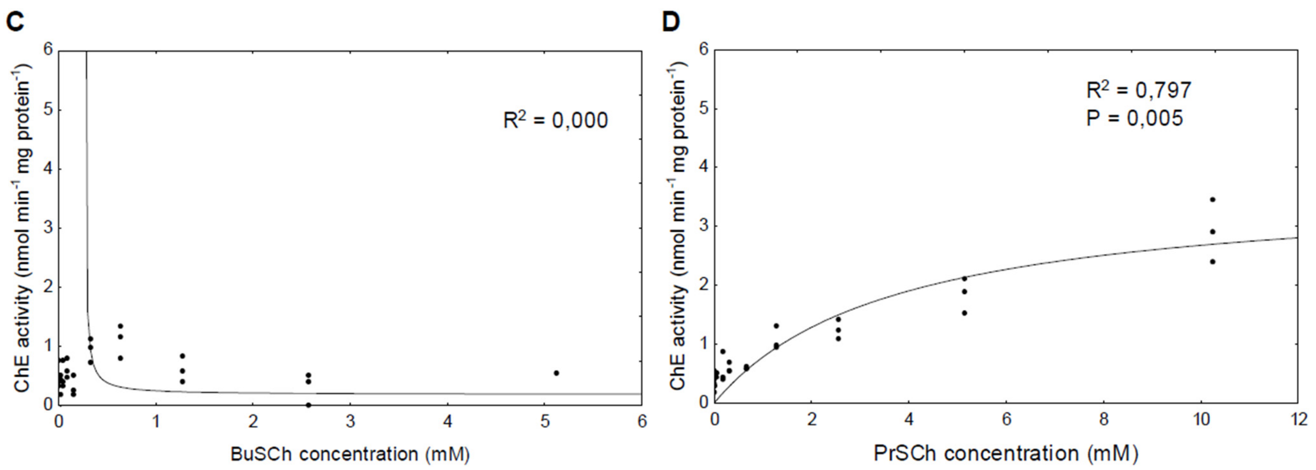

Figure S2. Hyperbolic regression of specific ChE activity of Pollicipes pollicipes capitulum soft tissues on substrate concentrations for each of the four substrates. (**A**): AcSCh; (**B**): AbSCh; (**C**): BuSCh; (**D**): PrSCh.

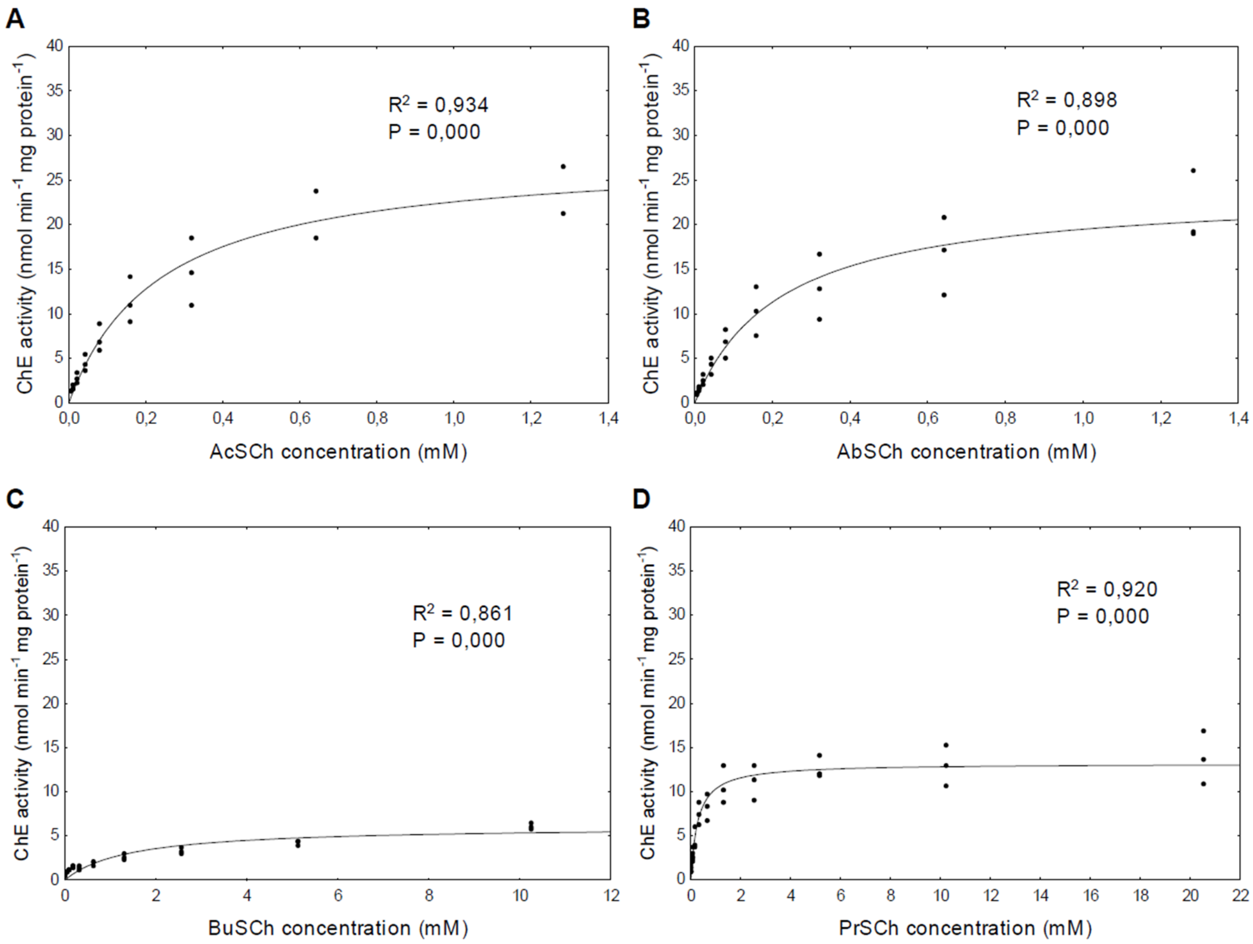

Figure S3. Hyperbolic regression of specific ChE activity of Perforatus perforatus soft tissues on substrate concentrations for each of the four substrates tested. (**A**): AcSCh; (**B**): AbSCh; (**C**): BuSCh; (**D**): PrSCh.
